# Optimization of Sequence, Display, and Mode of Operation of IgG-Binding Peptide Ligands to Develop Robust, High-Capacity Affinity Adsorbents That Afford High IgG Product Quality

**DOI:** 10.3390/ijms20010161

**Published:** 2019-01-04

**Authors:** Tuhidul Islam, Amith D. Naik, Yasuhiro Hashimoto, Stefano Menegatti, Ruben G. Carbonell

**Affiliations:** 1 Department of Chemical and Biomolecular Engineering, North Carolina State University, Raleigh, NC 27695-7905, USA; tuhin.acdu@gmail.com (T.I.); smenega@ncsu.edu (S.M.); 2 Biomanufacturing Training and Education Center (BTEC), North Carolina State University, Raleigh, NC 27695-7928, USA; amitnaik50@gmail.com; 3 Department of Research and Development, Fuji Silysia Chemical LTD, Kasugai Aichi 487-0013, Japan; y-hashimoto@fuji-silysia.co.jp

**Keywords:** affinity chromatography, peptide ligand, antibody purification, binding capacity, process optimization, aggregate removal

## Abstract

This work presents the use of peptide ligand HWRGWV and its cognate sequences to develop affinity adsorbents that compete with Protein A in terms of binding capacity and quality of the eluted product. First, the peptide ligand was conjugated to crosslinked agarose resins (WorkBeads) at different densities and using different spacer arms. The optimization of ligand density and display resulted in values of static and dynamic binding capacity of 85 mg/mL and 65 mg/mL, respectively. A selected peptide-WorkBeads adsorbent was utilized for purifying Mabs from Chinese Hamster Ovary (CHO) cell culture supernatants. The peptide-WorkBeads adsorbent was found able to withstand sanitization with strong alkaline solutions (0.5 M NaOH). The purity of the eluted product was consistently higher than 95%, with logarithmic removal value (LRV) of 1.5 for host cell proteins (HCPs) and 4.0 for DNA. HCP clearance was significantly improved by adding a post-load washing step with either 0.1 M Tris HCl pH 9 or 1 M NaCl. The cognate peptide of HWRGWV, constructed by replacing arginine (R) with citrulline, further increased the HCP LRV to 2.15. The peptide-based adsorbent also showed a remarkable performance in terms of removal of Mab aggregates; unlike Protein A, in fact, HWRGWV was found to bind only monomeric IgG. Collectively, these results demonstrate the potential of peptide-based adsorbents as alternative to Protein A for the purification of therapeutic antibodies.

## 1. Introduction

With over twenty products approved by the FDA and hundreds in clinical trials, monoclonal antibodies (Mabs) represent the dominant class of biotherapeutics, with a global market estimated at 140–200 billion dollars by 2022 [1]. These Mab products however are very expensive and unaffordable for people in emerging markets. The biopharmaceutical industry is facing a challenge to reduce the price through reduction in manufacturing costs. With the downstream processing accounting for 50–80% of the cost of goods (COGS) for Mabs [2,3] there is an interest to look into new alternative technologies and process improvements that can reduce the costs [4,5].

Several chromatographic and non-chromatographic techniques have been proposed for the purification of Mabs, fc fusion proteins and bispecifics [6,7,8,9] including efforts towards identifying alternatives to Protein A-based chromatography [10,11]. While being regarded as the standard for antibody purification owing to their high affinity and selectivity, these ligands present limitations, including high cost and the potential release of immunogenic fragments. An additional issue with Protein A concerns the presence of antibody aggregates in eluted fraction and their poor selectivity towards monomeric and correctly folded versus misfolded and/or aggregated IgG [12,13]. To overcome these limitations, a number of small synthetic ligands for IgG have been developed, including triazine scaffolds, nucleic acids, multimodal ligands, chelated metals, single amino acids, and peptides [14,15]. The latter class in particular—the peptide ligands—shows considerable promise as alternative to protein ligands [16,17,18]. Short peptides are generally nontoxic, chemically stable, and can be produced synthetically, hence relatively inexpensively, on the large scale, and with no batch-to-batch variability [19,20]. A number of IgG-binding peptides have been discovered over the last decade, including linear, branched, and cyclic sequences [21,22,23], and peptide mimetics [24,25,26]. Our group has identified a hexameric peptide—HWRGWV—that binds the Fc region of IgG [21,27], and extensively characterized its binding activity through experimental studies [21,28,29,30]. The objective of the present work was to enhance the dynamic binding capacity (DBC), impurity clearance capability and stability of the HWRGWV peptide ligand-based affinity media. 

There has been an increasing interest among the biotechnological industries to use carbohydrate chromatographic supports, such as agarose or cellulose, as these resins can be modified easily to attach ligand and exhibit low non-specific binding for host cell proteins [31]. Preliminary studies of coupling HWRGWV to an agarose base resin, WorkBeads ACT (Bio-Works, Uppsala, Sweden) had resulted in a higher IgG binding capacity as compared to polymethcarylate base resin [32]. In this work the peptide and peptide variants have been conjugated to Workbeads base resin at varying ligand densities and tested for binding capacity, selectivity and stability. 

The chromatographic affinity media are usually fabricated with a specific ligand covalently bonded to a support matrix through a spacer arm to increase the accessibility and flexibility of small ligands to reach the binding sites of target biomolecules. The spacer arms differ in chemical composition, length and degree of hydrophobicity [33,34,35]. Heldt et al. reported the influence of polyethylene oxide spacer arms on the binding of peptide ligands that capture porcine parvovirus [36]. In this study, spacer arms with different geometry (linear vs. branched), size, and hydrophilicity were screened to identify conjugation strategies that optimize the spatial orientation of the ligands, and hence their ability to interact with proteins in solution. Here, Tris(2-aminoethyl) amine (TREN), 3,3′-Diaminodipropylamine (DADPA), 3,3′-Diamino-*N*-methyldipropylamine (mDADPA), Hexamethylenediamine (HMDA), Ethylenediamine (EDA) and Polyethylene glycol (PEG) were evaluated as spacers. The chromatographic experiments were carried out at different process conditions to explore the effect of the composition and pH of the mobile phases on the overall performance of the ligand.

A major problem in the manufacturing of biopharmaceuticals using cell culture processes is the formation and removal of protein aggregates, composed of more than one molecule and containing completely or partially denatured molecules, which can occur from a variety of reasons and may involve both covalent and noncovalent interactions [37,38,39]. Protein aggregates in biotherapeutics can have contribution to serious immunogenicity as well as they can affect the activity of the therapeutic molecules in vivo [40,41,42]. The clinical consequences of anti-drug antibodies (ADA) in treated patients may vary widely, including anaphylaxis and infusion syndromes [43,44]. The presence of aggregates in the product stream eluted from Protein A media represents a known issue in antibody manufacturing. Protein A, in fact, is known to bind antibody aggregates present in the feed stream [37,45] and requires strong acidic elution conditions that drive further antibody aggregation. In this study, the Mab aggregate clearance capability of the peptide ligand was evaluated and compared to Protein A ligand. 

The proteolytic instability of protein and peptide ligand, especially the cleavage of peptide chains at the carboxyl side of the amino acids lysine or arginine, during the purification of immunoglobulins from animal plasma might adversely affect the binding activity of the ligands [46,47]. In consideration of these facts, as well as to reduce the ion-exchange interactions that contribute in binding BSA and HCPs [48,49], the arginine residue was replaced by an analogous non-natural and comparatively neutral amino acid, Citrulline. The chromatographic behaviors of the N-terminal acetylated variants were also examined. The influence of the spacer arms, process conditions, and peptide variants was determined by analyzing the chromatographic fraction through sodium dodecyl sulfate polyacrylamide gel electrophoresis (SDS-PAGE), Size-exclusion chromatography (SEC), enzyme-linked immunosorbent assay (ELISA), PicoGreen DNA quantitation and Dynamic Light Scattering (DLS).

## 2. Results and Discussion

### 2.1. Resin Fabrication

**Figure 1:**
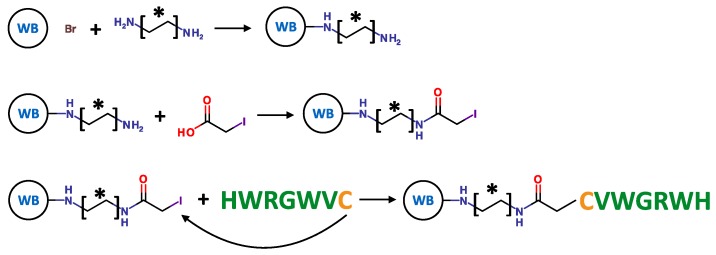
Fabrication of the HWRGWVC-WB resin by (**i**) substitution with an alkyl-amine spacer arm [-*-], (**ii**) activation with iodoacetic acid, and (**iii**) conjugation of the peptide ligand.

The agarose-based WorkBeads™ (WB) 40 ACT (functional density of ~250 µmol/mL) was incubated with 10× molar excess of each alkyl-amine spacer arm in water, overnight, at room temperature, and under mild stirring. The conjugation occurred by nucleophilic substitution between a primary amine group displayed by the alkyl-amine and the alkyl-bromide group displayed on the resin (Figure 1). The presence of primary amine groups on the WB resin was verified by Kaiser test [50]. The peptide ligands were subsequently immobilized to the aminated resin via iodoacetic acid (IAA) coupling chemistry as described in Section 3.2 (Figure 1). Briefly, IAA was activated with EDC and incubated with the WB resin, for 3 h, at room temperature, under mild stirring. The resulting iodoacetyl-WB resin was incubated with peptide HWRGWVC in DMF, overnight, at room temperature; the conjugation occurred by substitution between the thiol group displayed by the cysteine residue (C) on the HWRGWVC peptide and the iodoacetyl groups on the WB resin [32]. The supernatant was collected and analyzed by UV-Vis spectrophotometry at 280 nm to determine the amount of unconjugated peptide, from which the ligand density on the WB resin was derived. Unreacted iodoacetyl groups on the resin were saturated with 2-mercapto ethanol [32].

### 2.2. Effect of Spacer Arms

Numerous studies have investigated the effect of the physicochemical properties of the spacer arm (length, flexibility, and hydrophilicity) on the binding capacity and selectivity of the adsorbent [51,52,53]. The optimization of the spacer arm has been consistently found to improve the ligand display on the surface of the resin, and therefore the performance of the affinity adsorbent [35,36,54,55,56,57].

**Figure 2:**
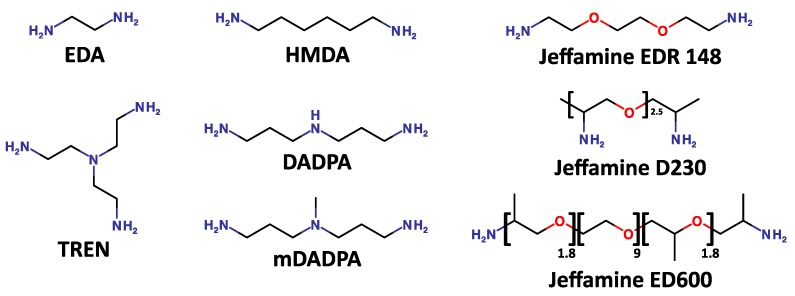
Structure of the spacer arms utilized in this work.

In this study, several di-/triamine spacers have been utilized (Figure 2), namely tris(2-aminoethyl) amine (TREN), 3,3′-diaminodipropylamine (DADPA), 3,3′-diamino-*N*-methyldipropylamine (mDADPA), hexamethylenediamine (HMDA), ethylenediamine (EDA), and polyethylene glycol (PEG)-based Jeffamines (EDR 148, D230 and ED 600). The effect of the spacer arms on the performance of the resin was evaluated by considering the dynamic binding capacity (DBC), purity, recovery, host cell protein (HCP) and DNA clearance. The PEG based spacer arms showed very low coupling to the resins as confirmed by Kaiser test. The ligand densities of the resins with TREN, DADPA, mDADPA, HMDA and EDA were 72, 63, 32, 57, 70 µmol/mL, respectively.

#### 2.2.1. Effect of Spacer Arm and Ligand Density on Dynamic Binding Capacity (DBC)

**Figure 3:**
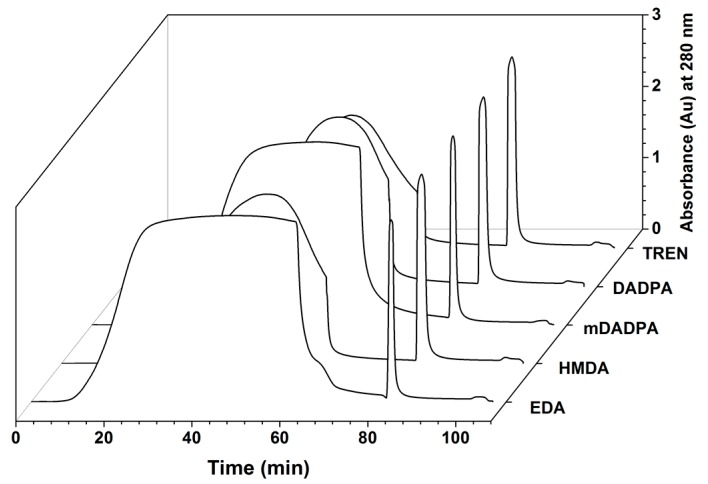
Breakthrough curves of the HWRGWVC-WB resins produced with different spacers.

The effect of the spacer arm on the dynamic binding capacity of HWRGWVC-WB resins was studied following the protocol described in Section 3.4. Based on the 10% breakthrough time read from the chromatograms in Figure 3 and the value of void volume measured via acetone pulse, the DBC for different adsorbents were calculated, and resulted as follows: 65 mg IgG/ mL adsorbent for TREN, 54 mg/mL for DADPA, 38 mg/mL for mDADPA, 51 mg/mL for HMDA, and 42 mg/mL for EDA (Table 1A). The adsorbents fabricated with the Jeffamine spacers did not bind IgG. This can be attributed to the low peptide density and nature of the spacer arm. While in fact a minimum length is required for the spacer arm to ensure the spatial outreach of the ligand, very long spacers like Jeffamines have a tendency to fold into random coils wherein the ligand is buried, thereby decreasing the accessibility of ligand for target binding. The high binding capacities obtained with TREN and DADPA indicate that a rigid spacer arm with an optimum length is required to orient the peptide ligand with high accessibility.

The effect of ligand density on binding capacity was also investigated. To this end, an ensemble of HWRGWV-TREN-WB adsorbents was produced with a range of ligand density viz. 72, 53, 37, and 22 µmol/mL and evaluated for DBC. As can be seen from Table 1B, values of DBC were found to be close, indicating that ligand display is the sole design parameter determining the behavior of peptide adsorbents in the entire range of ligand density explored.

#### 2.2.2. Effect of Spacer Arm on the Purity of Eluted IgG

**Figure 4:**
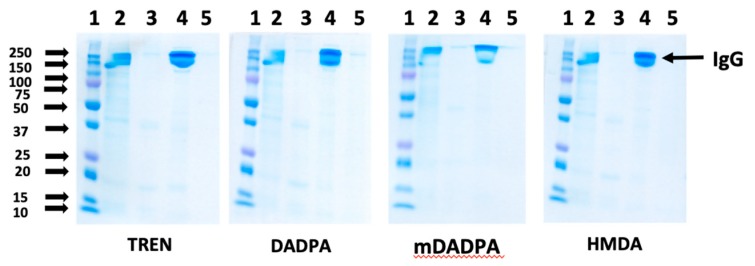
SDS-PAGE analysis (non-reducing conditions) of the chromatographic fractions generated by purifying Mab from a CHO cell culture supernatant using HWRGWV-WB resins constructed with different spacer arms (TREN, DADPA, mDADPA, and HMDA). Lanes: (1) Marker; (2) Load; (3) Flow-through; (4) Elution (pH 4.5); (5) Regeneration (pH 2.5).

To evaluate the effect of the spacer arm on the purity of the eluted product, the HWRGWV-WB adsorbents constructed with different spacer arms were utilized to purify a monoclonal IgG_1_ from a CHO cell culture supernatant. After loading, the column was washed with PBS, and the elution was performed with 0.1 M sodium acetate, pH 4.5. The collected fractions were analyzed by size exclusion chromatography to measure product yield (Table 2), and by SDS-PAGE to determine the overall product purity by densitometric analysis (Figure 4).

Notably, all the values of product yield (ratio of eluted vs. bound IgG) and purity were found to be higher than 90% regardless of the spacer utilized. Further, the chromatographic fractions were analyzed using a HCP ELISA kit and a Quant-IT Picogreen dsDNA assay kit to determine the levels of host cell proteins (HCPs) and DNA, respectively. The results reported in Table 2 indicate that the HWRGWV-mDADPA-WB resin, with an LRV of 1.35, gave the best HCP clearance. HWRGWV-HMDA-WB resin, on the other hand, showed the lowest HCP LRV, likely due to non-specific hydrophobic interactions between the HCP and the alkyl portion of the spacer arm. Notably, all the adsorbents consistently gave excellent values of DNA clearance.

### 2.3. Optimization of Process Conditions

While not providing the highest HCP clearance, the HWRGWV-TREN-WB resin showed a remarkably high binding capacity, and was therefore chosen as a model adsorbent for process optimization. The chromatographic conditions, viz., composition and concentration of the buffers, residence time during loading and flow rates, strongly influence the yield and purity of the recovered product. Therefore, identifying the optimal conditions is one of the main criteria for success in downstream bioprocessing.

**Figure 5:**
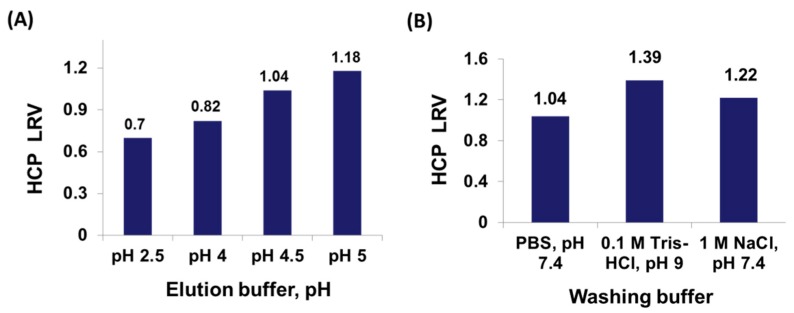
Values of HCP LRV obtained using different (**A**) elution buffers and (**B**) pre-elution wash buffers.

In this work, we have investigated the effect of pH of the elution buffer on the performance of HWRGWV-TREN-WB resin with a therapeutic monoclonal IgG_1_. Specifically, 0.1 M acetate buffer was used as a milder elution buffer (pH 4–5), while 0.1 M glycine HCl buffer was used for low pH elution (pH 2.5). The performance of the different buffers was evaluated in terms of HCP LRV in the eluted fraction, whose values are reported in Figure 5. The HCP clearance decreases at lower elution pH, which is however needed to ensure sufficient IgG yield. At pH 5, where a HCP LRV of 1.18 is obtained, only 75% of the bound IgG is eluted. At pH 4.5, the IgG yield increases to 95%, but the HCP clearance is lowered to 1.04. Finally, at pH 2.5, where the entirety of IgG is recovered, the HCP clearance drops to 0.7. Therefore, acetate buffer pH 4.5 was selected as elution buffer, as it offers the best combination of yield and purity.

These results indicate that HCPs are non-specifically bound by the ligand and co-elute with IgG, thereby affecting product purity. The majority of CHO HCPs are acidic in nature and have an overall negative charge at the physiological conditions (PBS pH 7.4) [58]. Their non-specific binding to the positively charged ligand (the net charge of HWRGWV at pH 7 is +1.1) is likely of electrostatic nature. In order to lower the non-specific capture of HCPs, pre-elution wash conditions were optimized by exploring different compositions and pH values of the washing buffer. Specifically, the following conditions were utilized, 0.2 M sodium phosphate pH 6, 0.2 M sodium phosphate pH 7, PBS pH 7.4, 1 M NaCl in PBS at pH 7.4, and 0.1 M Tris HCl pH 9. Two washing conditions, namely 0.2 M sodium phosphate at either pH 6 or 7, did not afford any HCP removal during washing. The addition of sodium chloride to the washing buffer, instead, was found to improve HCP clearance, from 1.04 (PBS, pH 7.4) to 1.22 (1 M NaCl in PBS, pH 7.4), whereas the highest HCP clearance was obtained with 0.1 M Tris HCl, pH 9, which afforded a HCP LRV of 1.39 (Figure 5).

**Figure 6:**
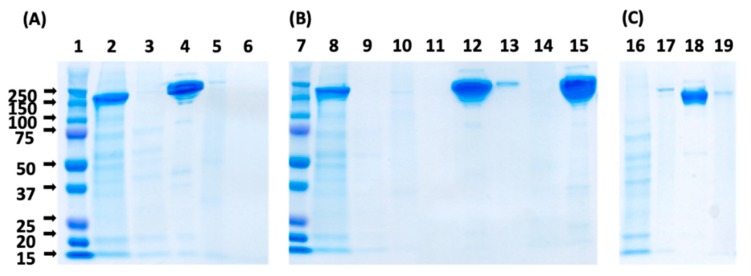
SDS-PAGE analysis of the chromatographic fractions generated by purifying IgG_1_ from a CHO cell culture supernatant using HWRGWVC-TREN-WB resins, with (**A**) no intermediate washing step (Lanes: (1) Marker; (2) Load; (3) Flow through; (4) Elution at pH 4.5; (5) Regeneration at pH 2.5; (6) NaOH cleaning); or (**B**) a washing step with 0.1 M Tris HCl, pH 9, included before elution (Lanes: (7) Marker; (8) Load; (9) Flow through; (10) Tris-HCl wash; (11) empty; (12) Elution at pH 4.5; (13) Regeneration at pH 2.5; (14) NaOH cleaning; (15) Standard IgG); and (**C**) a washing step with 1 M NaCl included before elution (Lanes: (16) Flow through; (17) 1 M NaCl wash; (18) Elution at pH 4.5; (19) Regeneration at pH 2.5).

As the pH reaches the isoelectric point of HWRGWVC (9.07), the ligands lose their positive charge and become electrically neutral, resulting in a weakening—or loss—of binding of the negatively charged HCPs. The SDS-PAGE analysis of the chromatographic fractions (Figure 6) indicates the inclusion of a washing step—with either 0.1 M Tris HCl, pH 9, or 1 M NaCl in PBS at pH 7.4—reduced the amount of impurities co-eluted with IgG. The use of NaCl, however, also causes a minor loss of IgG in the washing buffer, which is not observed when the Tris buffer is utilized. Therefore, 0.1 M Tris HCl, pH 9, was selected as optimal washing buffer. 

### 2.4. Studies on Ligand Variants

The studies presented above on the dependence of product yield and purity on the chromatographic conditions indicate a degree of non-specific binding of HCPs to the positively-charged peptide ligands. To minimize the positive charge of the peptide, we have investigated variants of HWRGWV constructed by replacing arginine (R) with citrulline (Cit) and acetylating the N-terminal histidine (H). Preliminary studies have shown improvement in the binding specificity of the ligand [46]. Therefore, in this work, the cognate peptides Ac-HWRGWVC, HWCitGWVC, and Ac-HWCitGWVC were tested in comparison with the original ligand HWRGWVC. 

#### 2.4.1. Adsorption Isotherm

**Figure 7:**
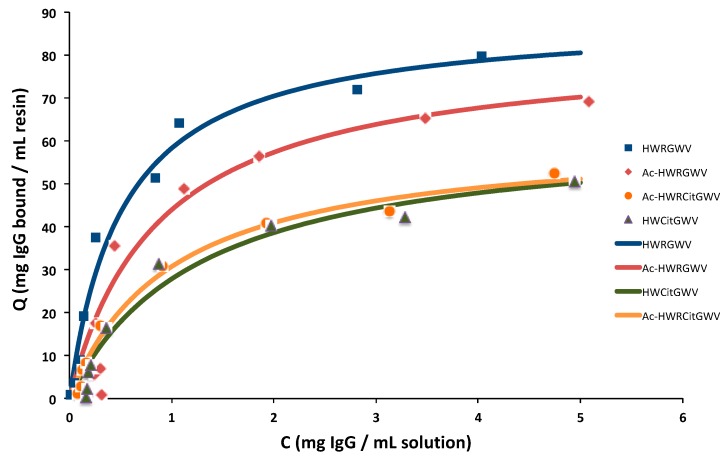
Adsorption isotherms of HWRGWV-TREN-WB, Ac-HWRGWV-TREN-WB, HWCitGWV-TREN-WB, and Ac-HWCitGWV-TREN-WB resins.

The IgG-binding affinity of the ligand variants was initially measured via batch adsorption studies. Briefly, aliquots of peptide-TREN-WB resin were incubated with solutions of IgG in PBS in a range of concentration between 0.1 and 12 mg/mL; as the binding equilibrium was reached, the amount of IgG adsorbed on each resin aliquot was calculated based on the equilibrium concentration of IgG in solution, and the corresponding values were fitted to the Langmuir model:

$$q=\frac{Q_{\max}C}{K_{D}+C}$$

where in *K_D_* is the dissociation constant and *Q*_max_ the equilibrium binding capacity. The resulting adsorption isotherms are shown in Figure 7, while the corresponding values of *K_D_* and *Q*_max_ are summarized in Table 3. Results indicate that the positively charged groups on the peptide ligand, i.e., the N-terminus and the arginine residue, play an important role in IgG binding. Replacing arginine with citrulline decreases the IgG-binding affinity (*K_D_*), and consequently the binding capacity of the adsorbent from 85 mg/mL to 49 mg/mL. Similarly, the acetylation of the peptide N-terminus consistently decreased both binding strength and capacity. 

#### 2.4.2. Purification of Mab Using Ligand Variants Constructed with Non-Natural Amino Acids

**Figure 8:**
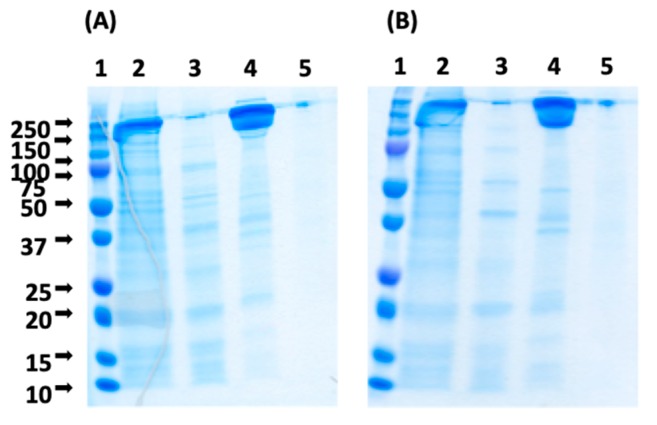
SDS-PAGE of the chromatographic fractions collected using (**A**) HWCitGWVC-TREN-WB and (**B**) Ac-HWCitGWVC-TREN-WB resins. Lanes: (1) Marker; (2) Load; (3) Flow-through; (4) Elution (pH 4.5); (5) Regeneration (pH 2.5).

While causing a decrease of binding capacity, the silencing of the positive charges on the peptide ligands, i.e., the N-terminus and arginine, lowers the non-specific binding of negatively charged protein impurities and improves product purity. Both HWCitGWVC-TREN-WB and Ac-HWCitGWVC-TREN-WB adsorbents were therefore tested by purifying monoclonal IgG_1_ from the CHO cell culture supernatant. After supernatant loading the column was washed with PBS and the elution was performed with 0.1 M sodium acetate at pH 4.5. The column was cleaned with aqueous 0.5 M NaOH, after which the purification run was repeated. The SDS-PAGE analysis of the chromatographic fractions is shown in Figure 8. The SEC analysis of the chromatographic fractions indicates that the purity of IgG eluted at pH 4.5 from HWCitGWVC-TREN-WB and Ac-HWCitGWVC-TREN-WB adsorbents was 94% and 97.5%, respectively. The HCP LRVs measured by ELISA were respectively 1.58 and 1.76. 

To further improve these results, purification runs with Ac-HWCitGWVC-TREN-WB adsorbent were carried out with intermediate washing steps using 1 M NaCl or 0.1 M Tris pH 9. As can be seen from Table 4 the wash steps increased HCP LRV considerably, with values of 2.07 and 2.15 for Tris pH 9 and 1 M NaCl respectively. This level of HCP reduction is the highest among the synthetic adsorbents reported in literature [23,24,60,61].

### 2.5. Removal of Aggregates

The removal of antibody aggregates is a major challenge in biomanufacturing. Antibody aggregation is triggered by a number of factors, such as temperature and pH variations, presence of oxidants, high total protein concentration in solution, and presence of misfolded proteins that act as aggregation triggers, and can occur at different segments of the production pipeline [37,38,39]. Protein aggregates are known to cause severe immunogenic response [40,41,42,43,44], and their clearance from therapeutic formulations is paramount [62,63,64]. The removal of misfolded or aggregated antibodies is made difficult by their low concentration and structural similarity with the target product. Protein A adsorbents have been shown to be unable to discriminate between monomeric and aggregated antibodies; further, the elution at low pH used during Protein A chromatography is known to trigger antibody denaturation and aggregation [65]. Antibody aggregates are currently removed after Protein A-based product capture using ion exchange chromatography, hydroxyapatite chromatography, or gel filtration, which suffers from low productivity due to limitations of low feed volume [66,67].

**Figure 9:**
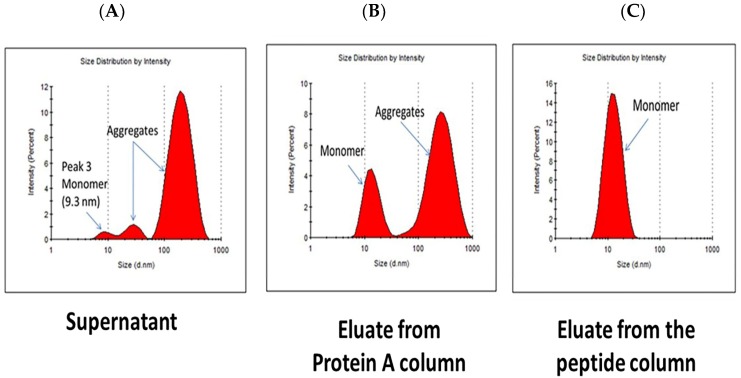
DLS analysis of (**A**) CHO cell culture supernatant, and the IgG-rich fraction eluted from (**B**) MabSelect SuRe LX and (**C**) HWRGWVC-TREN-WB resin. DLS measurements shown in Figure 9 are size distribution by intensity and therefore a small percentage of aggregates results in high peak area.

In this work, we have investigated the potential of the peptide-based adsorbent HWRGWVC-TREN-WB towards the separation of the Mab product from its aggregates, in comparison with MabSelect SuRe Lx resin. The presence of aggregates in the CHO cell culture supernatant was confirmed by dynamic light scattering (DLS) analysis using a Malvern Zetasizer µV (Figure 9A). It should be noted that the DLS measurements shown in Figure 9 are size distribution by intensity and therefore small percentage of aggregates results in high peak area. Resin equilibration and product binding was performed in PBS, pH 7.4, for both HWRGWVC-TREN-WB and MabSelect SuRe LX adsorbents, whereas elution was performed with 0.1 M sodium acetate buffer at pH 4.5 for the peptide resin and 0.1 M glycine HCl at pH 2.5 for the Protein A resin. The eluted fractions were neutralized with 0.5 M Tris HCl, pH 8, and analyzed by DLS to compare the ratio of monomeric vs. aggregated antibodies obtained with the different adsorbents (Figure 9B,C). Notably, the product eluted from the Protein A adsorbent was found to contain a significant amount of aggregates, as anticipated from previous studies [65,68], whereas no aggregates were detected in the fraction eluted from the HWRGWVC-TREN-WB resin (Figure 9).

**Figure 10:**
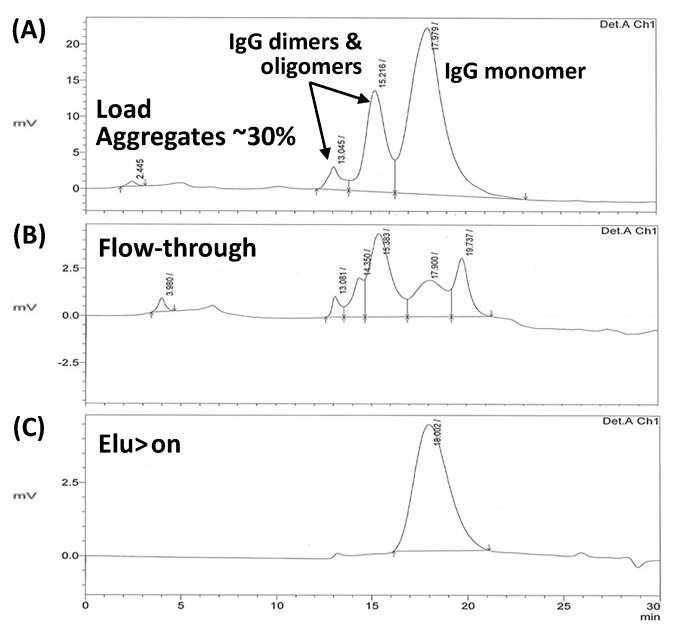
SEC-HPLC analysis of (**A**) Load on the peptide-WB column, polyclonal human IgG solution containing aggregates, (**B**) Flowthrough fraction from peptide-WB column and (**C**) Elution fraction from peptide-WB resin.

The ability of HWRGWVC-TREN-WB to remove aggregates was further tested by using a polyclonal IgG solution. The formation of aggregates was induced by incubating a 1 mg/mL IgG solution at 60 °C for 10 min. The IgG solution containing aggregates was loaded onto the HWRGWVC-TREN-WB resin, followed by washing with PBS and elution at pH 4.5. As it can be seen from the SEC analysis (Figure 10) of the samples the aggregates passed through the peptide column unbound while capturing only the monomeric form resulting in the elution fraction containing only monomeric IgG. 

The selectivity of HWRGWV to the monomeric form with respect to aggregates is likely due to its small size as compared to aggregated antibodies. As the DLS results indicate, the aggregates are mostly multimeric (more than two antibodies per aggregate). The size and complexity of such super-molecular structures (100–1000 nm) prevents the peptide ligand (Stokes’ radius ~0.75 nm) from effectively reaching its binding site on IgG. On the other hand, Protein A, owing to the combination of higher size (~5 nm) and IgG-binding strength, can effectively bind IgG in both its monomeric and aggregated form.

## 3. Materials and Methods

### 3.1. Materials

WorkBeads 40 ACT resin (WB) was purchased from Bio-Works Sweden AB (Uppsala, Sweden). MabSelect SuRe LX was received as a gift from GE Healthcare (Picastaway, NJ, USA). The peptides HWRGWVC, Ac-HWRGWVC, HWCitGWVC and Ac-HWCitGWVC in lyophilized form were purchased from Genscript (Piscataway, NJ, USA). The coupling agent 1-ethyl-3-(3-dimethylaminopropyl) carbodiimide (EDC) and iodoacetic acid were purchased from Acros Organics (Geel, Belgium). Ethylene diamine (EDA), tris(2-aminoethyl)amine (TREN), triethylamine (TEA), glycine, sodium acetate, sodium chloride, hydrochloric acid, phosphoric acid, anhydrous dimethylformamide (DMF), anhydrous acetone and ethanol were from Fisher Scientific (Pittsburgh, PA, USA). Hexamethylene diamine (HMDA), diaminodipropyl amine (DADPA), phosphate buffer saline (PBS), pH 7.4, and Kaiser test kit were from Sigma Aldrich (Saint Louis, MO, USA). Polyclonal Immunoglobulin G (IgG) in lyophilized form was purchased from Athens Research & Technology (Athens, GA, USA). Jeffamines (EDR148, ED600, D230) were a gift from Huntsman (The Woodlands, TX, USA). The clarified CHO cell culture supernatant contained a therapeutic IgG_1_ at 1.6 mg/mL. Mini-PROTEAN^®^ Tetra Vertical Electrophoresis Cell, Mini-Protein TGX precast gels (4–20%), Precision Plus (dual color) protein standard molecular marker, premixed 2× LaemmLi sample loading buffer, running buffer (10× Tris/Glycine/SDS), Bio-Safe™ Coomassie premixed staining solution, gel drying solution, GelAir dryer accessories were purchased from Bio-Rad (Hercules, CA, USA). AKTA Explorer from GE Healthcare (Picastaway, NJ, USA) and Waters 626 LC system integrated with 2487 UV detectors (Milford, MA, USA) were used for chromatography runs. HPLC was carried out on Agilent 1100 LC system equipped with an autosampler (Santa Clara, CA, USA). Omnifit EZ benchmark 6mm-ID /100mm-L columns were purchased from Kinesis Inc (Vernon Hills, IL, USA). Microbore PEEK columns 30 mm long × 2.1 mm I.D. were purchased from VICI Precision Sampling (Baton Rouge, LA, USA). The TSKgel G3000SW column was obtained from Tosoh Biosciences (Grove City, OH, USA). Invitrogen™ Quant-iT™ DNA Assay Kit was purchased from Fisher Scientific. CHO HCP ELISA kit (F550) was purchased from Cygnus Technologies (SE Southport, NC, USA). Fractions were analyzed by dynamic light scattering using Zetasizer μV (Malvern Panalytical, MA, USA).

### 3.2. Peptide Coupling onto the WorkBeads 40 ACT Resins

The diamine spacer arms were initially immobilized on WB 40 ACT resin (bromomethyl functional density of 250 µmol Br/mL resin) by nucleophilic substitution, and the peptide ligands were subsequently coupled by iodoacetate-based conjugation chemistry. In this work, ethylene diamine (EDA), tris(2-aminoethyl)amine (TREN), hexamethylene diamine (HMDA), diaminodipropyl amine (DADPA) and polyetheramines (Jeffamines EDR148, ED600, D230) were utilized. Briefly, each diamine spacer (10× molar excess as compared to the resin functional density) was incubated with 1 mL of a suspension of WorkBeads resin in water, overnight, at room temperature, and under mild stirring. Subsequently, a solution of 0.65 g of EDC and 0.8 g of iodoacetic acid (IAA) in 1 mL of water was prepared, its pH was adjusted to 4.5 using 50% HCl and 50% NaOH, and added to 1 mL of aminated resin. The reaction was allowed to proceed for 3 h, at room temperature, under mild stirring. The extent of IAA conjugation was monitored by Kaiser test. An amount of 0.1 g of peptide HWRGWVC was dissolved in 2 mL of anhydrous DMF with 10% *v*/*v* TEA, and the resulting solution incubated with 1 mL of iodoacetyl-WorkBeads resin. The coupling reaction was allowed to proceed overnight, in dark, at room temperature, and under mild stirring. The peptide density on the WorkBeads resin was determined by quantifying the unbound peptide by UV-Vis spectrophotometry at 280 nm. The unreacted iodoacetyl groups were saturated by 2-mercaptoethanol (50 µL) in 2 mL of DMF containing 10% (*v*/*v*) of TEA. The resin was rinsed and stored in 20% *v*/*v* ethanol at 4 °C.

### 3.3. Adsorption Isotherm Measurements

The adsorption of human polyclonal IgG by the HWRGWV-WB resins was measured at room temperature in a set of batch adsorption experiments. The resin was equilibrated with PBS, pH 7.4, and transferred into fritted tubes (50 µL of resin slurry per tube). Lyophilized IgG was dissolved in PBS, pH 7.4, at different concentrations ranging from 0.1 to 12 mg/mL. A volume of 1 mL of IgG solution was incubated with each of the resin aliquots for two hours, at room temperature, and under mild agitation. The supernatants were collected and quantified by UV-absorbance at 280 nm to determine the amount of IgG adsorbed by the resin and the corresponding equilibrium concentration of unbound IgG in solution, and the resulting data were fitted to a Langmuir isotherm.

### 3.4. Determination of Dynamic Binding Capacity

Thirty-five milligrams of peptide-WB resins, produced with different spacer arms and peptide ligand variants, were individually packed into microbore 30 mm × 2.1 mm I.D. columns (0.1 mL). The total void volume of the system was determined by an acetone pulse (1% *v*/*v*), as described in [30]. The resin was equilibrated with PBS, pH 7.4, and 1 mL of 10 mg/mL IgG in PBS was injected at the linear flow velocity of 35 cm/h (5 min residence time). Dynamic binding capacity was determined at 10% breakthrough. The resin was washed with PBS, and the elution was performed with 0.1 M Glycine HCl, pH 2.5. The flow velocity of washing and elution was 693 cm/h. 

### 3.5. Purification of IgG1 from CHO Cell Culture Supernatants

The peptide-WB resin (1 mL) was packed in an Omnifit column 6/100mm column and equilibrated with PBS. Sample of 10 mL of CHO cell culture supernatant was loaded onto the column at the linear flow velocity of 43 cm/h. After washing the resin with 4 CV of equilibration buffer, product elution was performed by 4 CV of 0.1 M sodium acetate buffer pH 4.5. Resin regeneration and cleaning was performed with 4 CV of 0.1 M glycine HCl pH 2.5 and 0.5 M NaOH, respectively. All the chromatographic steps after loading were performed at a flow rate of 106 cm/h. The chromatographic fractions were collected and analyzed to determine purity and yield. 

### 3.6. Determination of DNA Content

The chromatographic fractions were first diafiltered against 10 mM Tris–HCl pH 7.5, using Amicon^®^ centrifugal filters (3000 MWCO Millipore, Bedford, MA, USA). The DNA content in the fractions was determined by pico green assay using Quant-IT Picogreen dsDNA assay kit from Thermo Fisher Scientific (Philadelphia, PA, USA). Quantification was done as per the manufacturer’s protocol.

### 3.7. Determination of Host Cell Protein (HCP) Content

The host cell protein contents of the chromatographic fractions were determined using HCP ELISA kits from Cygnus Technologies (Southport, NC, USA). The high sensitivity protocol described by the manufacturer was used in the analysis. Briefly, the anti-CHO-coated microtiter wells were filled with 100 μL of standards and samples, and incubated on a rotator at 200 rpm for 1 h at room temperature. The wells were then washed three times with 300 μL washing buffer, and 100 μL of HRP-labeled anti-CHO antibody was added to each of the wells. After rinsing the wells, 100 μL of 3,3′,5,5′ tetramethyl benzidine (TMB) substrate solution was added to each well for 30 min at room temperature. The reaction was terminated by adding 100 μL of 0.5 M sulphuric acid. The amount of hydrolyzed substrate was measured by μQuant Microplate reader (BioTek Inc., Winooski, VT, USA) at 450 nm. A 4-parameter logistic fit was used to calculate the HCP (ng/mL) content in the chromatographic fractions. The HCP values determined in terms of ng/mL were converted into ng/mg of MAb to account for the dilution of fractions. The log reduction value (LRV) was determined as the log_10_ of the ratio of HCP in the load to the HCP in the elution fractions.

### 3.8. Aggregate Removal

Initially, two sets of fractions were produced by purifying IgG_1_ from the CHO supernatant using HWRGWVC-TREN-WB and MabSelect SuRe LX (Protein A) resins. PBS pH 7.4, was used as equilibration buffer, while elution was performed with 0.1 M acetate buffer pH 4.5 for HWRGWVC-TREN-WB resin, and with 0.1 M glycine HCl pH 2.5 for the MabSelect SuRe LX resin. The elution fractions were neutralized with 0.5 M Tris HCl pH 8 buffer and analyzed by dynamic light scattering (DLS) using a Zetasizer µV (Malvern Panalytical) to determine the presence and amount of antibody aggregates in feed sample, eluted fraction from HWRGWVC-TREN-WB resin, and eluted fraction from MabSelect SuRe LX resin.

Further, a feed sample containing monomeric and aggregated polyclonal IgG was produced by incubating a 1 mg/mL solution of human IgG at 60 °C for 10 min. The solution was loaded onto the HWRGWVC-TREN-WB resin, followed by washing with PBS and elution at pH 4.5. The collected fractions were analyzed by size exclusion chromatography (SEC) using a TSKgel G3000SW column to determine the ratio of monomeric vs. aggregated IgG in both flow-through and elution fractions.

## 4. Conclusions

This study presents a comprehensive characterization of a novel adsorbent, HWRGWVC-TREN-WB resin, to demonstrate the potential of peptide ligands as cost-effective affinity adsorbent for antibody manufacturing. To this end, we have optimized the adsorbent design, in terms of peptide sequence, display, and density, as well as the process conditions, in terms of composition and concentration of washing and elution buffers, in order to maximize both the binding capacity and the clearance of the three main classes of impurities, that is, HCPs, DNA, and aggregates.

Initially, the display of the peptide ligands on the surface of agarose-based WorkBeads resins (WB) was optimized using spacer arms, resulting in values of static and dynamic binding capacity (DBC) of about 85 mg/mL and 65 mg/mL, respectively. These values are unprecedented in the literature on synthetic affinity ligands [23,24,60,61]. The optimization of the peptide sequence and the chromatographic conditions enabled a noticeable increase of HCP clearance, from 1.04 obtained with HWRGWVC-TREN-WB, to 2.15 obtained with Ac-HWCitGWVC-TREN-WB coupled with a pre-elution wash with 1 M NaCl in PBS. While a high salt wash is not ideal for industrial application, the peptide-based adsorbents offer other significant advantages, such as the ability of eluting the product under mild conditions (pH 4.5) and the complete removal of antibody aggregates. The removal of aggregates in particular is recognized as a major concern in antibody purification. Another major advantage is represented by the innate stability of HWRGWV to alkaline CIP procedure. Identifying forms of Protein A capable to resist alkaline cleaning and sanitization over a high number of cycles has required an extensive process of protein engineering, and the best performing adsorbents are currently on the market at $8000–15,000 per liter. On the other hand, HWRGWV-based adsorbents are stable to alkaline treatment and can be fabricated relatively inexpensively, owing to the low cost of large-scale peptide synthesis.

Collectively, these studies present a method for developing peptide-based adsorbents to be employed in Biomanufacturing and demonstrate their effectiveness as compared to traditional protein-based media.

## Figures and Tables

**Table 1:** Effect of (**A**) spacer arms and (**B**) ligand density on the DBC of HWRGWVC-TREN-WB.

(A)			(B)	
Spacer	Ligand Density (μmol/mL Adsorbent)	DBC (mg·IgG/mL Adsorbent)	Ligand Density on TREN-WB (μmol/mL Adsorbent)	DBC (mg·IgG/mL Adsorbent)
**TREN**	72	65	22	61
**DADPA**	63	54	37	64
**mDADPA**	32	38	53	64
**HMDA**	57	51	72	65
**EDA**	70	42	-	-

**Table 2:** Values of product yield and purity obtained for a monoclonal IgG_1_ purified from a CHO cell culture supernatant using HWRGWV-WB resins constructed with different spacer arms.

Spacer	Yield (%)	HCP LRV	DNA LRV
TREN	97	1.04	4
DADPA	96	1.16	4
HDMA	98	0.97	4
EDA	94	1.12	4
mDADPA	93	1.34	4

**Table 3:** Values of dissociation constant (*K_D_*) and the equilibrium binding capacity (*Q*_max_) obtained from fitting the IgG adsorption data of HWRGWV-TREN-WB, Ac-HWRGWV-TREN-WB, HWCitGWV-TREN-WB, and Ac-HWCitGWV-TREN-WB resins to a Langmuir model. Protein A-based adsorbent MabSelect SuRe, is reported for comparison.

Ligand	*Q*_max_ (mg·IgG/mL Adsorbent)	*K_D_* (µM)
HWRGWV	85	1.04
Ac-HWRGWV	72	1.16
HWCitGWV	49	0.97
Ac-HWCitGWV	50	1.12
Protein A [59] (MabSelect SuRe)	77	-

**Table 4:** Values of HCP and DNA clearance obtained with four Peptide-TREN-WB adsorbents under different washing conditions. Elution was performed with 0.1 M Na-acetate at pH 4.5.

Ligand	Washing Buffer	HCP LRV	DNA LRV
HWRGWV	PBS, pH 7.4	1.04	~4
HWCitGWV	PBS, pH 7.4	1.58	~4
Ac-HWRGWV	PBS, pH 7.4	1.38	~4
Ac-HWCitGWV	PBS, pH 7.4	1.76	~4
Ac-HWCitGWV	1 M NaCl in PBS, pH 7.4	2.15	~4
Ac-HWCitGWV	0.1 M Tris HCl, pH 9	2.07	~4

## Abbreviations

CHO	Chinese hamster ovary
DADPA	diaminodipropyl amine
DCM	dichloromethane
DIPEA	diisopropylethylamine
DMF	*N*,*N*-dimethylformamide
EDA	ethylene diamine
EDC	1-ethyl-3-(3-dimethylaminopropyl) carbodiimide
EDT	ethandithiol
Fmoc	fluorenylmethyloxycarbonyl
HMDA	hexamethylene diamine
IAA	iodoacetic acid
IgG	Immunoglobulin G
PBS	phosphate buffer saline
SDS-PAGE	sodium dodecyl sulfate polyacrylamide gel electrophoresis
TEA	triethylamine
TREN	tris(2-aminoethyl)amine
WB	WorkBeads 40 ACT resin
